# HIV Replication Increases the Mitochondrial DNA Content of Plasma Extracellular Vesicles

**DOI:** 10.3390/ijms24031924

**Published:** 2023-01-18

**Authors:** Wilfried Wenceslas Bazié, Julien Boucher, Benjamin Goyer, Dramane Kania, Isidore Tiandiogo Traoré, Diane Yirgnur Somé, Michel Alary, Caroline Gilbert

**Affiliations:** 1 Axe de Recherche Maladies Infectieuses et Immunitaires, Centre de Recherche du CHU de Québec-Université Laval, Québec, QC G1V 4G2, Canada; 2 Programme de Recherche sur les Maladies Infectieuses, Centre Muraz, Institut National de Santé Publique, Bobo-Dioulasso 01 BP 390, Burkina Faso; 3 Institut Supérieur des Sciences de la Santé, Université Nazi Boni, Bobo-Dioulasso 01 BP 1091, Burkina Faso; 4 Axe de Recherche Santé des Populations et Pratiques Optimales en Santé, Centre de Recherche du CHU de Québec-Université Laval, Québec, QC G1S 4L8, Canada; 5 Département de Médecine Sociale et Préventive, Faculté de Médecine, Université Laval, Québec, QC G1V 0A6, Canada; 6 Institut National de Santé Publique du Québec, Québec, QC G1V 5B3, Canada; 7 Département de Microbiologie-Infectiologie et d’Immunologie, Faculté de Médecine, Université Laval, Québec, QC G1V 0A6, Canada

**Keywords:** extracellular vesicles, HIV-1, mitochondrial DNA, antiretroviral therapy, tenofovir, zidovudine

## Abstract

Extracellular vesicles (EVs) and their cargo have been studied intensively as potential sources of biomarkers in HIV infection; however, their DNA content, particularly the mitochondrial portion (mtDNA), remains largely unexplored. It is well known that human immunodeficiency virus (HIV) infection and prolonged antiretroviral therapy (ART) lead to mitochondrial dysfunction and reduced mtDNA copy in cells and tissues. Moreover, mtDNA is a well-known damage-associated molecular pattern molecule that could potentially contribute to increased immune activation, oxidative stress, and inflammatory response. We investigated the mtDNA content of large and small plasma EVs in persons living with HIV (PLWH) and its implications for viral replication, ART use, and immune status. Venous blood was collected from 196 PLWH, ART-treated or ART-naïve (66 with ongoing viral replication, ≥20 copies/mL), and from 53 HIV-negative persons, all recruited at five HIV testing or treatment centers in Burkina Faso. Large and small plasma EVs were purified and counted, and mtDNA level was measured by RT-qPCR. Regardless of HIV status, mtDNA was more abundant in large than small EVs. It was more abundant in EVs of viremic than aviremic and control participants and tended to be more abundant in participants treated with Tenofovir compared with Zidovudine. When ART treatment was longer than six months and viremia was undetectable, no variation in EV mtDNA content versus CD4 and CD8 count or CD4/CD8 ratio was observed. However, mtDNA in large and small EVs decreased with years of HIV infection and ART. Our results highlight the impact of viral replication and ART on large and small EVs’ mtDNA content. The mechanisms underlying the differential incorporation of mtDNA into EVs and their effects on the surrounding cells warrant further investigation.

## 1. Introduction

Analysis of extracellular vesicles (EVs) is emerging as a diagnostic tool for tailoring therapeutic strategies for chronic diseases. Based on their biogenesis, these membranous secretions are classified as exosomes, microvesicles, or apoptotic bodies [1,2]. They are shed by most cell types as a means of exchanging material and information both locally and remotely [1,2]. As active carriers of specific proteins, lipids, microRNA, DNA, cytokines, chemokines, and even cell receptors, they constitute biomarkers of physiological status [1,2], useful for early diagnosis and monitoring of cancer progression and predicting treatment outcomes [3,4]. The EV fraction of blood or lymph is often regarded as a liquid biopsy [3,4]. The role of EVs in the pathogenesis of human immunodeficiency virus (HIV) infection has been highlighted [5,6,7,8]. Based on their contents, they have been implicated both in viral replication and in the immune response to infection [9,10]. However, one category of their molecular cargo, namely, mitochondrial DNA (mtDNA), remains largely unexplored in the context of HIV infection, even though it is well known that HIV infection and prolonged antiretroviral therapy lead to mitochondrial dysfunction [11].

Mitochondria are specialized organelles that play a major role in energy production for cell processes [12]. They have an autonomous circular genome consisting of double-stranded DNA. Human cells contain 100 to 1000 copies of mtDNA, which represent less than 1% of their total nucleic acid content. Mitochondria are involved in the regulation of numerous cellular functions including proliferation, apoptosis, intracellular calcium homeostasis, and immune responses [13,14]. Since they play an active role in several fundamental processes, their dysfunction affects a wide range of cellular functions and can cause a variety of diseases [15].

Accumulating evidence suggests that mitochondria in persons living with HIV (PLWH) exhibit a dysfunctional state marked by altered mitochondrial membrane potential, reactive oxygen species accumulation, decreased adenosine triphosphate generation, loss of mass, and depletion of mtDNA [11]. Decreased mtDNA copy number per cell is a well-known side effect of some antiretroviral therapy (ART) drugs. Classes of ART approved for the treatment of HIV include nucleoside-analog reverse transcriptase inhibitors (NRTIs), non-nucleoside reverse transcriptase inhibitors (NNRTIs), protease inhibitors (PIs), integrase inhibitors (INIs), fusion inhibitors, and coreceptor antagonists, each of which interferes with critical steps in the viral replication lifecycle [16,17]. NRTIs are the most common drug class to be incorporated in first line ART regimen. They interfere in the polymerization by being incorporated into viral DNA and cause chain termination to disrupt the viral capacity to complete reverse transcription of viral RNA into DNA [17,18]. NRTIs were known to inhibit DNA polymerase gamma (Pol-γ) responsible for mtDNA synthesis and leading to mtDNA depletion [11,19,20,21]. This has been observed notably in adipocytes [22] and in nerve cells, which has been proposed as the cause of mitochondrial neuropathy [23], in peripheral blood mononuclear cell (PBMC) and in plasma [24,25,26]. This inhibitory activity varies widely among the various molecules of INRTIs [11,20,27,28]. Alternatively, mitochondrial dysfunction is also associated with NNRTIs, PIs, and INIs, despite these drug classes not disrupting Pol-γ activity [11,16].

Mitochondrial DNA can be liberated by active, stressed, apoptotic, or necrotic cells and circulate as cell-free DNA [29]. Mitochondrial DNA packaged in EVs is reportedly transferred between cells [30,31], and circulating mtDNA has been found significantly more concentrated in EVs than in the associated plasma fraction [32,33]. Transfers of EV-borne mtDNA to cells has been shown to restore energy production in metabolically impaired cells [34]. Mitochondrial DNA is recognized as part of a damage-associated molecular pattern and, therefore, could potentially activate pattern recognition receptors such as Toll-like receptors (TLRs) 9, absent in melanoma 2 (AIM-2), and cyclic GMP-AMP Synthase (cGAS) [35]. EV-borne mtDNA could contribute directly to increased immune activation, oxidative stress, and inflammatory response, since it is un-methylated and resembles bacterial DNA [35,36,37]. This functionality supports the hypothesis that EV mtDNA content may shed light on the immune activation and inflammation seen in persons living with HIV.

To investigate the possible relationship between EV mtDNA content and the health of PLWH, we purified the large and small EVs fractions in plasma of HIV-infected and uninfected participants, as we have published previously [6,8,38], and compared these with viral replication; use of NRTI, NNRTIs, and protease inhibitors; and immune status.

## 2. Results

### 2.1. Mitochondrial DNA Is More Abundant in EVs of ART-Naive Viremic Patients

We analyzed the mtDNA inside large and small EVs of all participants samples described in Table 1 and Figure S1. The data shown in Figures S2 and S3 summarize the quality controls of the EV preparations. The purification method allowed the separation of two populations of vesicles with an average size (*n* = 249) of 309 ± 155 nm for large EVs and 130 ± 39 nm for small ones (*p* < 0.0001). As observed by previous authors, the large EVs were more susceptible to lysis by detergents than small EVs (Figure S2C).

**Figure 1:**
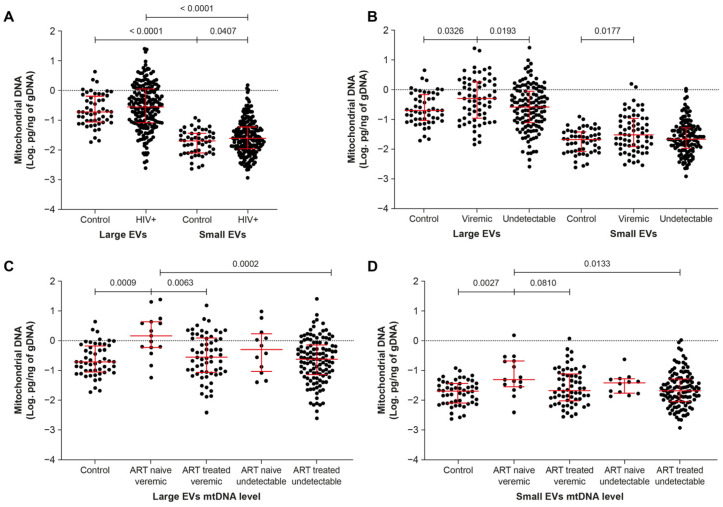
Mitochondrial DNA content of large and small EVs in viremic and non-viremic participants. (**A**) HIV-infected versus uninfected participants; (**B**) in viremic and non-viremic participants irrespective of antiretroviral therapy (ART); (**C**,**D**) in viremic and non-viremic participants on ART or not. In graphs, the dots represent individual value and lines the geometric mean with geometric standard deviation factor. Groups were compared using *t*-tests and ordinary one-way ANOVA with Tukey’s multiple comparisons test.

Regardless of participant HIV status, mtDNA was more abundant in large than in small EVs (*p* < 0.0001, Figure 1A and Figure S3B, Table S1). Its range of abundance was broader in small EVs of PLWH than in those of uninfected participants (Figure 1A) and it was generally more abundant in male than female PLWH, independent of viremia (Figure S3D–F).

Having determined that mtDNA was more abundant in large EVs, we then investigated the effect of HIV infection status, viral replication, and ART on the mtDNA content of large and small EVs. Irrespective of ART, large EVs carried more mtDNA in viremic than aviremic and control participants, and small-EV-borne mtDNA was more abundant in viremic than control participants (Figure 1B). Large-EV-borne mtDNA was more abundant in viremic ART-naive than viremic ART-treated participants (Figure 1C). No significant difference was noted between ART-treated viremic and control participants, in small or large EVs (Figure 1C,D). In small EVs, no significant difference was observed between viremic and aviremic ART-naive participants, although mtDNA tended to be more abundant in the viremic group compared with the control and ART-treated virus undetectable groups (Figure 1D). These observations collectively highlight an over-abundance of mtDNA in EVs, mainly in ART-naive participants, suggesting involvement of HIV replication in enrichment of EVs with mtDNA.

### 2.2. ART including Tenofovir Increases the mtDNA Content of EVs

**Figure 2:**
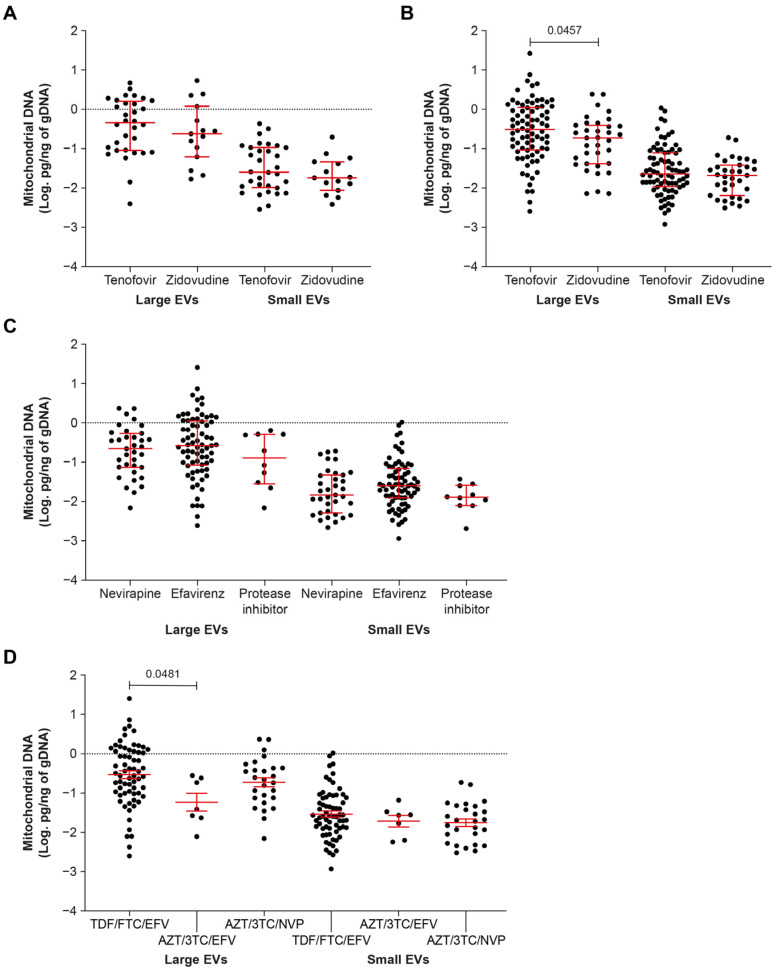
Mitochondrial DNA in large and small EVs in viremic (**A**) and aviremic (**B**) patients on antiretroviral therapy including Zidovudine or Tenofovir for more than six months; in aviremic patients receiving Nevirapine, Efavirenz, or protease inhibitors (**C**); or a combination of Tenofovir/Emtricitabine/Efavirenz (TDF/FTC/EFV), Zidovudine/lamivudine/Efavirenz (AZT/3TC/EFV), or Zidovudine/lamivudine/Nevirapine (AZT/3TC/NVP) (**D**). In graphs, the dots represent individual value and lines the geometric mean with geometric standard deviation factor. Group comparisons are based on *t*-tests and ordinary one-way ANOVA with Tukey’s multiple comparisons test.

Depletion of mtDNA in PBMC, plasma, and tissues has been associated with ART [22,23,24,25,26]. In patients treated with NRTI drugs for more than six months, we observe that Tenofovir increases mtDNA in large EVs compared with Zidovudine in aviremic (Figure 2B). Whether Zidovudine or Tenofovir was used, no significant difference was observed between viremic and aviremic participants (Figure S4A,B).

NNRTI drugs, Nevirapine or Efavirenz, and protease inhibitors (Darunavir or Lopinavir boosted by Ritonavir) had no significant differential effect on mtDNA in large or small EVs (Figure 2C). Significantly more mtDNA was found in large EVs in participants taking Tenofovir + Emtricitabine + Efavirenz than in those taking Zidovudine + Lamivudine + Efavirenz (Figure 2D). No difference between first line and second line NRTI treatments in combination with NNRTI drugs and protease inhibitors was observed for large or small EVs (Figure S4D). These results overall show a trend towards mtDNA enrichment in large EVs of Tenofovir-treated persons living with HIV.

### 2.3. EV-Borne mtDNA Abundance Appears Indifferent to CD4 and CD8 T Cell Counts

**Figure 3:**
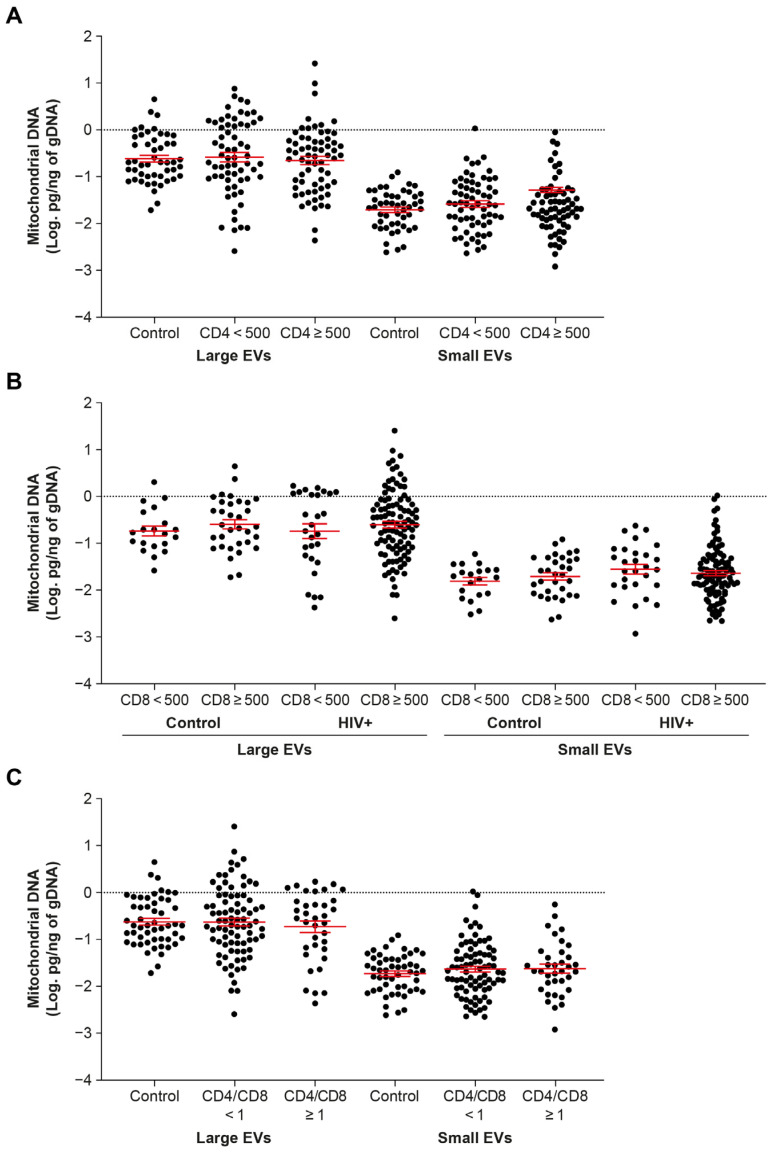
Mitochondrial DNA content of large and small EVs in individuals in different categories of CD4 T cell count (**A**), CD8 T cell count (**B**), and CD4/CD8 ratio (**C**). In graphs, the dots represent individual value and lines the mean with the standard error of the mean. Ordinary one-way ANOVA with Tukey’s multiple comparisons was used to reveal significant differences.

Levels of mtDNA in persons living with HIV reportedly vary with CD4 and immune activation markers [24,25,26]. In this study, participants on ART for more than six months and not viremic were split into groups with CD4 or CD8 T cell counts <500 and ≥500, and CD4/CD8 ratios <1 and ≥1. This made no difference to the amount of mtDNA found in large or small EVs (Figure 3A–C). Furthermore, correlational analysis between these and other clinical parameters (HIV status, viremia, ART) showed no significance for large EVs (Table S2) or small EVs (Table S3).

### 2.4. Men Who Have Sex with Men Have More Small-EV-Borne mtDNA

**Figure 4:**
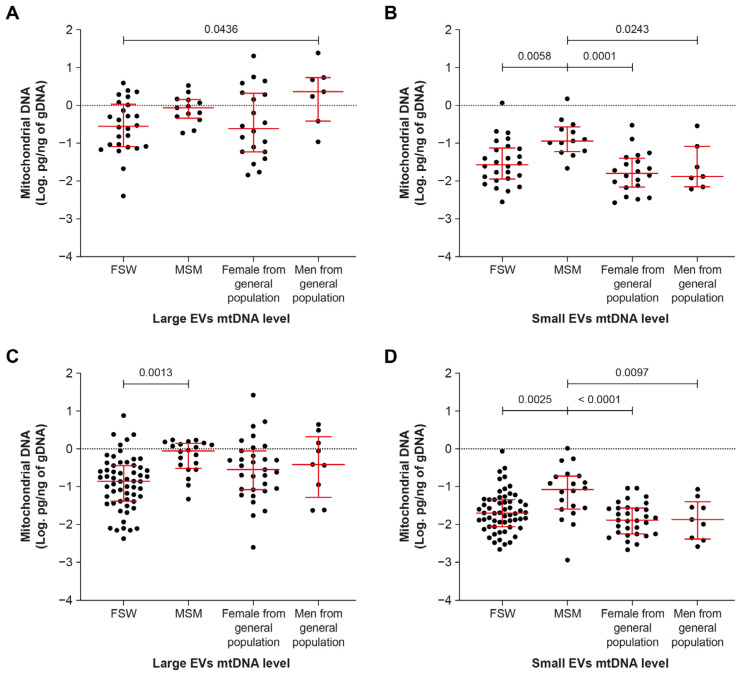
Mitochondrial DNA content of large and small EVs in HIV-infected participant subgroups. (**A**,**B**) viremic participants; (**C**,**D**) aviremic participants. FSW—female sex workers; MSM—men who have sex with men. In graphs, the dots represent individual value and lines the geometric mean with geometric standard deviation factor. Ordinary one-way ANOVA with Tukey’s multiple comparisons test was used to reveal significant differences between groups.

The amount of mtDNA in EVs appears to differ among the categories of participants in this study (Figure 4). It was significantly higher in small EVs in men who have sex with men (MSM) compared with other groups regardless of viremic status (Figure 4B,D).

**Figure 5:**
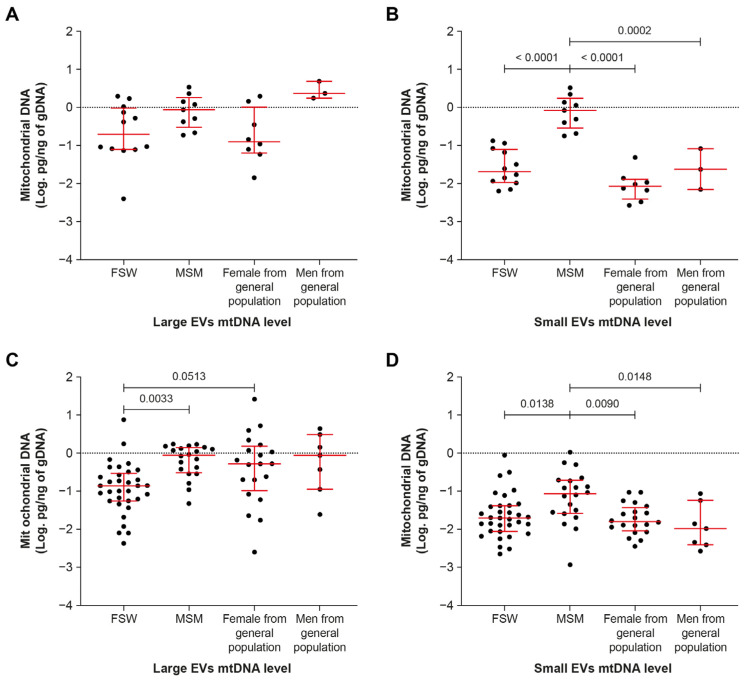
Mitochondrial DNA abundance in large and small EVs in viremic (**A**,**B**) and non-viremic (**C**,**D**) Tenofovir-treated participants. FSW—female sex workers, MSM—men who have sex with men. In graphs, the dots represent individual value and lines the geometric mean with geometric standard deviation factor. An ordinary one-way ANOVA with Tukey’s multiple comparisons test was used to reveal significant differences between groups.

The group of MSM contained mostly Tenofovir-treated younger males with fewer years of infection and ART treatment (Table 2 and Table 3). Refocusing the analysis on Tenofovir-treated participants, a similar trend was observed—that is, more mtDNA in small EVs regardless of viremia (Figure 5B,D). Regarding whether this increase in mtDNA is related to an increase in the number of EVs, no difference was observed in small EV counts between the viremic and non-viremic ART-treated groups (Figures S5 and S6).

**Figure 6:**
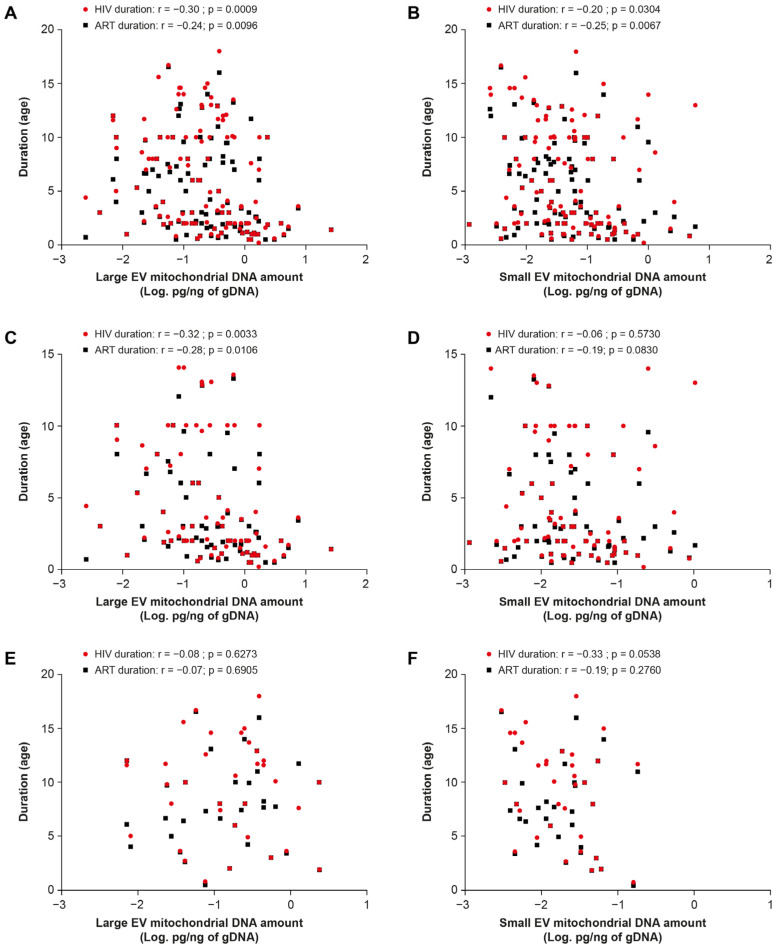
Pearson analysis of correlations between mtDNA abundance in large and small EVs of non-viremic participants and years of HIV+ status or years on ART. (**A**,**B**) ART without NRTI drugs; (**C**,**D**) Tenofovir-treated participants; (**E**,**F**) Zidovudine-treated participants.

Based on our analyses, mtDNA levels in large and small EVs were inversely correlated with age, years since HIV infection, and years on ART (Figure 6A,B; Tables S2 and S3). Moreover, the correlation coefficient was stronger for years since infection and on ART than for age. The correlation with years on ART was significant in Tenofovir-treated but not Zidovudine-treated participants (Figure 6C,D). These results collectively suggest a relationship between the mtDNA content of EVs and years since HIV infection or on ART as well as the ART molecule used.

### 2.5. The mtDNA Content of Large EVs Can Distinguish HIV+ Viremic Patients

**Figure 7:**
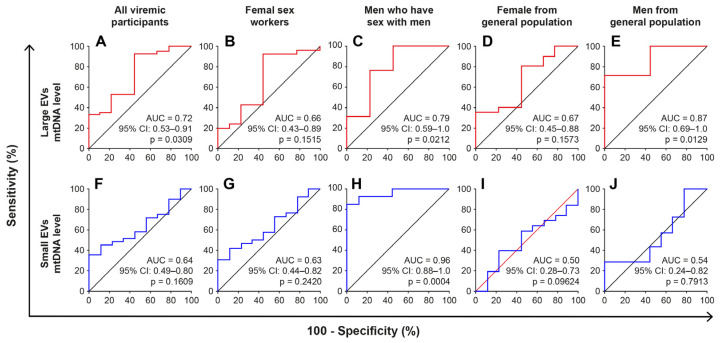
Performance of the mtDNA content of large and small EVs as a discriminator of viremic HIV+ patients, based on receiver operating characteristic curve analysis. Large and small EVs mtDNA level was used to generate a Receiver Operator Characteristic (ROC) curve analysis to discriminate viremic participants. Participants with <500 CD8 T cells/µL, ≥500 CD4 T cells/µL, and CD4/CD8 ratio ≥ 1 (group 1, *n* = 10) were used as controls. Diagnosis performance of large EVs and small EV-mtDNA level for the discrimination of all viremic participants (**A**,**F**), viremic female sex workers (**B**,**G**), viremic men who have sex with men (**C**,**H**), and viremic women (**D**,**I**) and men (**E**,**J**) from the general population respectively. The Wilson/Brown method was used to compute the area under the curve.

Having observed that EV-borne mtDNA is more abundant in viremic participants and given that mtDNA is considered pro-inflammatory, we used receiver operator characteristic curve analysis to test the performance of mtDNA as an indicator of patients with ongoing viral-replication or immune activation, which is characterized by a CD4/CD8 ratio <1 and a CD8 T cell count >500 cells/µL, as described previously [38]. The control group was ART-treated aviremic participants with ≥500 CD4 T cells/µL and <500 CD8 T cells/µL. EV-borne mtDNA allows discrimination of viremic participants, with an area under the curve of 0.72 (95% CI 0.53–0.91) for large EVs and 0.64 (95% CI 0.49–0.80) for small EVs (Figure 7 and Table S4). The test performed best in the MSM group, with an area of 0.79 (95% IC 0.59–1.0) for large EVs and 0.96 (95% IC 0.88–1.0) for small EVs.

Using the CD4/CD8 ratio and CD8 T cell count thresholds, the test result was significant only in the MSM group, with an area under the curve of 0.72–0.83. No significant results were observed for any other participant/patient subgroup (Table S4). Based on these results, the potential usefulness of microvesicular mtDNA as a biomarker for monitoring relatively recent HIV infection cannot be ruled out.

## 3. Discussion

It has been reported previously that at least 90% of circulating non-cell-associated mtDNA is extracellular vesicle borne [33,39]. In this study, we compared the mtDNA content of purified large and small plasma EVs from persons living with HIV and from uninfected participants. We found that large plasma EVs carry more mtDNA regardless of HIV status and even more so in the case of participants with ongoing HIV replication, particularly in ART-naive viremic patients.

The release of microvesicles carrying mitochondrial DNA, proteins, and other contents and even of whole mitochondria by several cell types after activation has been reported [32,40,41]. Transfers of large microvesicles but not of exosomes reportedly leads to increased mitochondrial function in hypoxic endothelial cultures [42]. This is consistent with our observation that mtDNA was more abundant in large than in small EVs. Since large and small EVs have different subcellular origins and likely target different cell types for different physiological purposes, their contents should be expected to differ.

Although mtDNA was more abundant in large EVs of viremic participants, its abundance had no significant correlation with viremia. Little is known about EV-borne mtDNA levels during HIV infection and even less about its distribution among plasma EV subtypes. Plasma mtDNA has been found significantly more abundant during acute HIV infection and in late presenters taking antiretroviral therapy for the first time than in healthy individuals or in long-term non-progressors, and a positive correlation with plasma viral load has been observed [43]. More divergent results have been obtained in other studies [24,26], and mtDNA has been found to be inversely associated with HIV RNA [24,26]. These divergences may be due to the heterogeneity of the populations studied, methodology, specific HIV infection state, and coinfections. It is known that viral infection alters cell physiology and function and can directly or indirectly impair mitochondrial function and dynamics to facilitate viral proliferation [44,45]. The mtDNA content of large EVs appears to distinguish viremic patients with fair reliability. Further studies will be necessary to determine the molecular mechanisms underlying the enrichment of large EVs with mtDNA in viremic patients.

Among viremic participants, mtDNA was more abundant in large and small plasma EVs of ART-naive patients. Evidence is mounting that stress can trigger mtDNA release from the mitochondria to the cytosol or extracellular space, but how this occurs remains unclear [29]. The presence of HIV proteins (Env, Tat, Nef, and Vpr) inside cells during viral replication appears to induce cytochrome c release and activate the intrinsic apoptotic pathway as well as cause outer membrane permeabilization [11,46]. An increase in mitochondrial membrane permeability could allow extrusion of mtDNA into the cytosol and hence passive incorporation into EVs. Indeed, HIV-replication-induced impairment of the mitophagy pathway [47] can lead to accumulation of damaged mtDNA and its release into the cytoplasm through mitochondrial membrane pores. The damaged mtDNA can be further released to extracellular space from the cell by several ways including through EVs under oxidative stress or virus infection [29,48,49,50,51].

Despite viral replication, ART seems to decrease EV-borne mtDNA. These results suggest that ART somehow decreases cell mtDNA content or incorporation of mtDNA into EVs during HIV infection. It was found that after treatment with NRTIs, mitochondrial dysfunction emerges due to mtDNA depletion [52,53]. NRTIs inhibit Pol-γ, the mtDNA replication enzyme in eukaryotic cells, which functions in mtDNA replication and maintenance, implicating ART as a potential cause of mitochondrial dysfunction [11,16,52,54]. NRTIs inhibit Pol-γ through four different mechanisms encompassing their effects as follows: (i) mtDNA chain terminators (once incorporated into a growing strand, DNA replication is abruptly halted); (ii) competitive inhibitors (competing with natural nucleotides to be incorporated into growing DNA chains by polymerase gamma); (iii) inductors of errors in the fidelity of mtDNA replication inhibiting the exonucleolytic proofreading function of polymerase gamma); (iv) contributors to the decrease in mtDNA reparatory exonuclease activity (resisting exonucleolytic removal by exonuclease activity of polymerase gamma because of the lack of the 3OH group in NRTIs) [54,55]. The NRTI toxicity may be dose-dependent and cumulative, and toxic manifestations increase with duration of exposure [56,57].

However, not all NRTIs contribute to mtDNA depletion. In vitro studies reported a hierarchy of mtDNA depletion for various NRTIs. Maximum inhibition was observed after treatment with Zalcitabine followed by Didanosine and Stavudine. Lamivudine, zidovudine, and abacavir were found to be weak inhibitors of gamma polymerase [20]. In our study, participants treated with Tenofovir showed a trend of having increased amounts of mtDNA in large EVs compared with Zidovudine. Similarly, with regard to the ratio of mtDNA to nuclear DNA in PBMC obtained from HIV post-exposure prophylaxis in uninfected patients, Bañó et al. reported that at 6 months of follow-up, while the ratio decreased in the Zidovudine-treated group compared with baseline, it increased in the Tenofovir-treated patients [58]. Tenofovir is a nucleotide analogue reverse transcriptase inhibitor with a weak capacity to inhibit polymerase-γ in vitro [57,59,60]. It has been reported that Tenofovir at its maximal concentration has no discernable effect on cell count, lactate production, mtDNA content, the mtDNA-encoded respiratory chain subunit II of cytochrome c oxidase (COXII) expression, or intracellular lipids [27,56,57]. However, Zidovudine-induced cytotoxicity increased when Lamivudine or Emtricitabine were also given, even though neither of these cytidine analogs alone were more than mildly toxic [27,56,57]. The Pol-γ inhibition associated with Zidovudine in combination with other molecules in the long term [19,61] could lead to variable reductions of mtDNA in cells and, consequently, in EVs, such as we observed. Our findings highlight questions such as would Tenofovir induce the production of a new class of mitochondrial DNA-rich EVs and how does the absence of mtDNA toxicity translate into mtDNA enrichment of EVs? The mechanisms explaining mtDNA incorporation into EVs of Tenofovir patients deserve to be elucidated in experimental studies.

We found an inverse correlation between EV-borne mtDNA and duration of HIV+ status or ART use and a weak inverse correlation with age among HIV+ participants. The duration of ART would be a confounding factor of the HIV status duration. A decline in plasma EV mtDNA with age has been noted previously [33]. Long-term exposure to HIV and ART has been associated with chronic mitochondrial dysfunction [61] and explains easily the age-related decline seen in our study. The combined effects of younger age, fewer years of infection, and Tenofovir use appear sufficient to explain the greater abundance of EV-borne mtDNA in the MSM group. No other characteristic of this group emerges as an explanation.

We observed no correlation between EV-borne mtDNA and CD4 or CD8 T cell count or CD4/CD8 ratio among ART-treated non-viremic participants, unlike some studies in which plasma mtDNA appeared to be associated directly with CD4 T cell counts [24,26]. In another study, no association was observed between platelet mtDNA and CD4 count, CD4 nadir, or CD4/CD8 ratio in long-term ART-treated persons living with HIV [43,62]. This lack of coherence between EV-borne mtDNA with HIV infection and its established clinical monitoring parameters suggests considering other markers, particularly cytokines, in assessing the EV-borne mtDNA as possible biomarkers of the immune activation and inflammation seen during HIV infection.

There are some limitations to our study. Given the cross-sectional design, one cannot determine causality or the direction of the relationship (Did viral replication or Tenofovir lead to increased mtDNA quantity in EVs?). In addition, the size of some groups is small and would contribute to under or overestimating the significance of some analyses. Despite these methodological limitations, our results provide information that should be taken into account in further analysis of EVs in the context of HIV infection.

In conclusion, our results showed the differential enrichment of EV subpopulations with mtDNA and the impact of viral replication or ART on this measurement. The mechanisms underlying the differential incorporation of mtDNA into EVs and their effects on surrounding cells or tissues warrant further investigation. The discovery of new biomarkers for monitoring the health of persons living with HIV remains an important challenge, and interest in the possible contribution of EV analysis (size distribution, abundance, and content) is growing. We believe that a longitudinal study of EV-borne mtDNA analysis taking into consideration the limitations of this study will lead to a better understanding of the mechanism, the implications of its concentration, and possibly its use as a biomarker for improved management of HIV infection.

## 4. Materials and Methods

### 4.1. Study Participants

HIV-infected patients (*n* = 196) were recruited by referral from 5 follow-up centers in Bobo-Dioulasso and Ouagadougou (Burkina Faso). These included the Yerelon clinics (Centre Muraz) of both towns, specialized in female sex worker follow-up; the Association African Solidarité (AAS) center for the follow-up of men who have sex with men (MSM); and dedicated follow-up day hospital centers—Centre Hospitalier Universitaire (CHU), Souro Sanou (Bobo-Dioulasso), and CHU Yalgado Ouédraogo (Ouagadougou). The HIV-negative participants (*n* = 53) were recruited in the Yerelon clinics, which also offer HIV testing.

All subjects were anonymous volunteers and provided written informed consent to participate in the study.

### 4.2. Quantitation of HIV-1 RNA, CD4 and CD8 T Lymphocytes

The HIV-1 viral load was measured using the COBAS^®^ AmpliPrep /COBAS^®^ TaqMan^®^ Real-Time PCR assay (TaqMan, Roche Diagnostics, Mannheim, Germany), which targets two highly conserved regions of the HIV-1 genome and has a detection limit of 20 copies/mL.

Absolute counts of CD4^+^ T and CD8^+^ T lymphocytes were obtained using a BD FACSCount™ System flow cytometer (Becton Dickinson, San Jose, CA, USA).

### 4.3. Purification of Extracellular Vesicles

EVs were purified as described previously [6,7,8]. Briefly, blood obtained by venipuncture with citrate as an anticoagulant was centrifuged for 10 min at 400× *g* at room temperature. The plasma was centrifuged for 10 min at 3000× *g* to obtain platelet-free-plasma and stored at −80 °C until analysis. Thawed platelet-free plasma (250 µL) was treated with proteinase K (1.25 mg/mL, Ambion™, Thermo Fisher Scientific, Waltham, MA, USA) for 10 min at 37 °C, which reduces substantially the amount of non-EV protein (albumin, apolipoproteins A-1 and B) and RNA that otherwise remains present. The plasma was then centrifuged at 17,000× *g* at room temperature to pellet large EVs. The supernatant was mixed with 63 µL of ExoQuick (SBI via Cedarlane, Burlington, ON, Canada) in an Eppendorf tube and maintained at 4 °C overnight. The large EV pellet was resuspended in 250 µL of microfiltered (0.22 µm pore size membrane) 1× phosphate-buffered saline (PBS) (WISENT Bioproducts, Saint-Jean-Baptiste, QC, Canada) and centrifuged for 30 min at 17,000× *g*. This supernatant was discarded, and the washed large EV pellet was resuspended in 250 µL of PBS and kept at 4 °C. Small EVs were pelleted as ExoQuick precipitate by centrifuging for 30 min at 1500× *g*, resuspended in PBS, centrifuged for 5 min at 1500× *g* (both supernatants were discarded) and then in 250 µL of PBS by vortex mixing, and kept at 4 °C. All relevant experimental data have been submitted to the EV-TRACK knowledgebase (EV-TRACK ID: EV220124) [63]. Characteristics of the purified EVs are summarized in Figure S2.

### 4.4. EV Size Measurement

EV size was estimated as hydrodynamic radius using a Zetasizer Nano S (Malvern Instruments, Ltd., Malvern, UK), which measures light scattering due to Brownian motion at a fixed position with an automatic attenuator and controlled temperature. The size distribution is obtained from the diffusion constants using the Einstein–Stokes equation [64]. Two measurements on 100 µL samples of EV suspension were averaged (Figure S2A,B).

### 4.5. EV Flow Cytometry Analysis

Purified EVs were stained with the lipophilic fluorescent carbocyanine dye DiD (DiIC18(5) solid or 1,1′-dioctadecyl-3,3,3′,3′-tetramethylindodicarbocyanine 4-chlorobenzene sulfonate salt, Invitrogen™, Carlsbad, CA, USA) and the vesicular or cell-permeable dye CFSE (carboxyfluorescein diacetate succinimidyl ester, Invitrogen™, Carlsbad, CA, USA). DiD and CFSE were diluted, respectively, 1/100 and 1/500 in filtered (0.22 µm) PBS 1X + EDTA to 100 µM. Diluted DiD solution was then mixed with EV suspension (40 µL and 10 µL, respectively, for a final DiD concentration of 1 µg/mL). After 5 min at 37 °C, 50 µL of CFSE were added (final CFSE concentration 1 µg/mL). After 15 min (37 °C), the staining was fixed by adding 0.02% Pluronic F-127 (Invitrogen™, Carlsbad, CA, USA) solution, followed by 100 µL of 4% paraformaldehyde (Fisher Scientific™, Ottawa, ON, CA), holding for 20 min, and then adding 200 µL of filtered PBS 1X (final volume 400 µL). Count bead suspension (Polybead^®^ microspheres, 15 µm, Polysciences, Inc., Warrington, PA, USA) was mixed in (5 µL) by vortex. EVs were counted using a flow cytometry method described previously [6,7,8] in a FACS Canto II Special Order Research Product cytofluorometer equipped with forward scatter coupled to a photomultiplier tube (FSC-PMT) with the “small particles option” (BD Biosciences, Franklin Lakes, NJ, USA). Gating strategies for EV identification and analysis are described elsewhere [8].

### 4.6. Detergent Treatment of Extracellular Vesicles

To evaluate the true nature of large and small EVs fraction, we performed a detergent control analysis with Sodium dodecyl sulfate (SDS) (WISENT Bioproducts, Saint-Jean-Baptiste, QC, Canada) and Triton 100-X (Fisher Scientific™, Ottawa, ON, CA). For this, isolated EVs were stained with DID and Cell Trace Violet Cell Proliferation kit (Invitrogen™, Eugene, OR, USA) as performed for CFSE. After the fixation of staining with 0.02% Pluronic F-127, EVs were supplemented with SDS solution for final concentration in 200 µL of 0.025%, 0.075%, and 0.150%. For Triton 100-X, the final concentrations were 0.025% and 0.075%. These conditions were established based on previous studies [65] and preliminary experiments. The mixture was vortexed for 30 s and held for 30 min at room temperature. Then, 100 µL of 4% paraformaldehyde (Fisher Scientific™, Ottawa, ON, CA), holding for 20 min, and adding 100 µL of filtered PBS 1X (final volume 400 µL) before flow cytometry analysis.

### 4.7. DNA Extraction and Mitochondrial DNA Quantification

A mixture of EV suspension (100 µL) plus lysis buffer (475 µL of 0.4% *m*/*v* Tris base, 1% *m*/*v* sodium dodecyl sulfate, and 100 mM EDTA) plus proteinase K (25 µL, 20 mg/mL) was held at 55 °C for 10 min. UltraPure™ phenol/chloroform/isoamyl alcohol (25:24:1 by volume, Invitrogen, Carlsbad, CA, USA) was added (500 μL), followed by vigorous shaking then centrifuging at 12,000× *g* for 3 min at 4 °C. The aqueous phase (350 μL) was shaken with an equal volume of chloroform and centrifuged at 12,000× *g* for 3 min at 4 °C. The aqueous supernatant (200 μL thereof) was mixed thoroughly with 20 µL of 5.0 M NaCl, then 400 μL of ice-cold absolute ethanol and 1 μL of GlycoBlue™ co-precipitant (Invitrogen™, Carlsbad, CA, USA), and held at −20 °C for 1 h. After centrifugation for 20 min at 12,000× *g* at 4 °C, the supernatant was removed and the pellet was washed with 700 μL of cold 70% ethanol, followed by centrifugation at 12,000× *g* for 8 min. Pellets were dried at room temperature and resuspended in 15 μL of TE 1X Buffer (WISENT Bioproducts, Saint-Jean-Baptiste, QC, Canada). The DNA was held at 4 °C overnight and quantified in 1 µL aliquots using a BioDrop-μLITE kit (Isogen Life Science, Utrecht, The Netherlands).

Quantitative real-time PCR was performed using a CFX384 Touch Real-Time PCR Detection System (Bio-Rad, Hercules, CA, USA) with a SYBR^®^ Green PCR kit (Qiagen, Hilden, Germany) and specific primers. The targeted genes included human mitochondrial NADH dehydrogenase subunit 5. The primer sequences (Integrated DNA Technologies) were forward ACGCCTGAGCCCTATCTATTA-3′ (Tm = 54.9) and reverse 5′-GTTGACCTGTTAGGGTGAGAAG-3′ (Tm = 55). The amplification temperature profile was as follows: 15 min at 95 °C (enzyme activation) then 40 cycles of 15 s at 94 °C (denaturation), 30 s at 55 °C (annealing), 30 s at 72 °C (elongation). Reaction specificity was confirmed using the melt curve procedure (65–95 °C, 0.5 °C per 5 s) at the end of the amplification according to the manufacturer’s instructions.

A standard curve for mtDNA copy quantification was generated using total DNA obtained from human platelets and diluted in series. All samples were analyzed in triplicate, and a no-template control was included in every analysis. Quantity of mtDNA was expressed in picograms per nanogram of total DNA.

### 4.8. Transmission Electron Microscopy

Briefly, EV pellets, large or small, purified as described above, were mixed with 2.5% glutaraldehyde (Sigma-Aldrich, St. Louis, MO, USA) plus 0.1 M sodium cacodylate (Sigma-Aldrich, St. Louis, MO, USA) buffer pH 7.4, pipetted (10 µL) onto a nickel grid with carbon-coated formvar film, and held for 10 min. Excess liquid was removed by blotting. The grids were stained for 2 min with 2% uranyl acetate solution. Images were acquired at 80 kV using a FEI Tecnai Spirit G2 (FEI, Eindhoven, The Netherlands) transmission electron microscope equipped with a bottom-mounted CCD camera.

### 4.9. Statistical Analysis

All analyses were performed with GraphPad Prism 9.4.1 (GraphPad Inc, San Diego, CA, USA). Participant demographic and clinical characteristics were presented as a proportion or median with an interquartile range (IQR) and tabulated (Table 1). Initial tests of normality and log normality indicated that EV count and mtDNA content in picograms fit a lognormal distribution; therefore, all values were transformed to logarithms. Data were then analyzed assuming a Gaussian distribution using parametric tests, and ordinary one-way ANOVA corrected for multiple comparisons using the Tukey test for three or more group comparisons. All graphed results are presented as the geometric mean with geometric standard deviation factor or mean with standard error of mean. Pearson parametric correlation tests were performed. The diagnostic value of the EV miRNA content was evaluated using receiver operating characteristic curves. Analyses were performed using the Wilson/Brown method, and the results were tabulated. In Table 1, the Chi-Square Test (if applicable) or Fisher’s test was used for the comparison of categorical variables and a *t*-test for continuous variables. A *p* value less than 0.05 was considered statistically significant.

## Figures and Tables

**Table 1:** Characteristics of the study participants.

	HIV–Control (Never Infected) *n* = 53	HIV+ *n* = 196	*p* Value	HIV+ ART > 6 Months, VL Undetectable *n* = 118	HIV+ ART-Naïve, VL Undetectable *n* = 12	*p* Value	HIV+ ART > 6 Months, VL Detectable *n* = 51	HIV+ ART-Naïve, VL Detectable *n* = 15	*p* Value
Male: *n* (%)	14 (26.4)	54 (27.5)	0.8692	29 (24.6)	5 (41.67)	0.1993	14 (27.45)	6 (40.00)	0.3525
Female sex workers: *n* (%)	-	85 (43.4)	-	58 (49.1)	1 (8.3)	0.0002	24 (47.1)	2 (13.3)	0.0870
Men who have sex with men: *n* (%)	-	33 (16.8)	-	20 (16.9)	0 (0.0)		9 (17.6)	4 (26.7)	
Age in years, median (IQR)	25 (22–32)	37 (31–44)	<0.0001	38 (32–44)	43 (28–52)	0.2072	35 (30–42)	35 (26–42)	0.4629
HIV+ duration, median months (IQR)	-	43 (22–121)	*-*	50 (24–120)	24 (12–72)	0.1229	43 (24–117)	8 (1–114)	0.4643
CD4 T cells/µL, median (IQR)	937 (800–1316)	473 (340–696)	<0.0001	492 (368–750)	717 (466–875)	0.1057	372 (188–507)	632 (304–798)	0.0691
CD8 T cells/µL, median (IQR)	564 (451–717)	757 (535–1011)	<0.0001	739 (536–1089)	650 (398–759)	0.1245	856 (544–1016)	791 (556–1016)	0.4482
CD4/CD8 ratio, median (IQR)	1.8 (1.4–2.1)	0.6 (0.4–1.0)	<0.0001	0.7 (0.5–1.0)	1.2 (0.7–1.5)	0.0028	0.4 (0.2–0.7)	0.7 (0.5–1.2)	0.0120
ART, *n* (%)	-	169 (86.22)		118 (100)	0		51 (100)	0	
NRTI ART drugs, *n* (%)								-	
Abacavir	-	7 (4.1)		3 (2.5)	-		4 (7.8)	-	
Tenofovir	-	112 (66.3)		80 (67.8)	-		32 (62.7)	-	
Zidovudine	-	50 (29.6)		35 (29.7)	-		15 (29.4)	-	
NNRTI ART drugs or PI, *n* (%)	-								
Efavirenz	-	104 (61.5)		73 (61.9)	-		31 (60.8)	-	
Nevirapine	-	46 (27.2)		35 (29.7)	-		11 (21.6)	-	
Protease inhibitors	-	19 (11.2)		10 (8.5)	-		9 (17.6)	-	
Months on ART, median (IQR)	-	36 (21–88)		38 (21–92)	-		24 (21–69)	-	
HIV undetectable (<20 copies/mL) while on ART, *n* (%)	-	118 (69.8)		-	-		-	-	
HIV load, median copies/mL (IQR)	-	2.841 (82–29.240)		-	-		1.606 (77–22.510)	13.174 (2.297–70.711)	0.2451

ART: antiretroviral therapy, CD4: CD4 T cell count, CD8: CD8 T cell count, HIV: human immunodeficiency virus, IQR: interquartile range, NRTI: nucleoside reverse transcriptase inhibitors, NNRTI: non-nucleoside reverse transcriptase inhibitors, VL: viral load.

**Table 2:** Characteristics of non-viremic participants.

	Female Sex Workers (*n* = 58)	Men Who Have Sex with Men (*n* = 20)	Females from General Population (*n* = 31)	Males from General Population (*n* = 9)	*p* Value
Age, median (IQR)	38 (33–44)	30 (25–38)	42 (35–46)	45 (45–46)	0.0003
CD4 T cell count, median (IQR)	507 (372–825)	485 (351–565)	543 (434–758)	295 (265–455)	0.0759
CD8 T cell count, median (IQR)	748 (569–1075)	559 (430–653)	822 (590–1206)	751 (540–856)	0.1768
Years HIV+, median (IQR)	6.0 (3.1–10.0)	1.7 (1.0–4.0)	7.1 (2.0–12.8)	2.0 (1.5–7.0)	0.0108
Years on ART, median (IQR)	4.6 (2.6–8)	1.7 (1.1–2.2)	5.3 (2.0–10.0)	1.0 (0.9–6.6)	0.0021
On Tenofovir, *n* (%)	33 (56.9)	20 (100)	20 (64.5)	7 (77.8)	0.0012
Large EV count, log_10_ EVs/µL plasma, median (IQR)	3.6 (3.4–3.8)	3.7 (3.6–3.8)	3.7 (3.4–4.1)	4.2 (4.0–4.3)	0.0006
Small EV count, log_10_ EVs/µL plasma, median (IQR)	4.0 (3.9–4.4)	4.0 (3.8–4.1)	3.9 (3.8–4.4)	4.3 (3.8–4.4)	0.1540

IQR: interquartile range; HIV: human immunodeficiency virus.

**Table 3:** Characteristics of viremic participant subgroups.

	Female Sex Workers (*n* = 26)	Men Who Have Sex with Men (*n* = 13)	Females from General Population (*n* = 20)	Males from General Population (*n* = 7)	*p* Value
Age, median (IQR)	35 (33–41)	25 (25–29)	40 (33–44)	35 (25–49)	<0.0001
CD4 T cell count, median (IQR)	394 (266–550)	395 (321–514)	465 (176–703)	208 (164–563)	0.4819
CD8 T cell count, median (IQR)	883 (641–1013)	770 (438–913)	729 (543–1196)	630 (480–1147)	0.2414
Viral copies/mL, median (IQR)	2432 (90–38,126)	5442 (98–19,481)	3632 (72–34,053)	1563 (186–35,481)	0.6147
Years HIV+, median (IQR)	3.7 (2.0–10.8)	0.8 (0.5–2.1)	6.0 (3.3–13.0)	1.7 (0.8–18.8)	0.0053
ART-treated, *n* (%)	24 (92.3)	9 (69.2)	13 (65.0)	5 (71.4)	0.0869
Years on ART, median (IQR)	3.4 (2.0–7.2)	1.0 (0.7–2.0)	5.0 (2.4–7.5)	0.7 (0.7–6.1)	0.0385
On Tenofovir, *n* (%)	12 (50)	9 (100)	8 (61)	3 (60)	0.0908
Large EV count, log_10_ EVs/µL plasma, median (IQR)	3.8 (3.6–4.0)	3.9 (3.8–4.1)	4.1 (3.5–4.2)	4.3 (4.1–4.5)	0.0375
Small EV count, log_10_ EVs/µL plasma, median (IQR)	3.9 (3.8–4.4)	3.9 (3.8–4.0)	3.9 (3.8–4.5)	4.2 (3.8–4.5)	0.6392

IQR: interquartile range; HIV: human immunodeficiency virus.

## Data Availability

De-identified participant data from this study and corresponding data dictionary, study protocol, and informed consent documents will be made available to researchers upon request to the corresponding author. Researchers will be asked to complete a concept sheet for their proposed analyses to be reviewed, and the investigators will consider the overlap of the proposed project with active or planned analyses and the appropriateness of study data for the proposed analysis.

## Supplementary Materials

The following supporting information can be downloaded at https://www.mdpi.com/article/10.3390/ijms24031924/s1.
