# Supplementary material for: HIV Replication Increases the Mitochondrial DNA Content of Plasma Extracellular Vesicles
Source: Int J Mol Sci. 2023 Jan 18;24(3):1924. doi: 10.3390/ijms24031924 (PMC9916095; doi:10.3390/ijms24031924)
Supplement: Supplementary file 1 [file ijms-24-01924-s001.zip › ijms-2111589-supplementary.pdf]

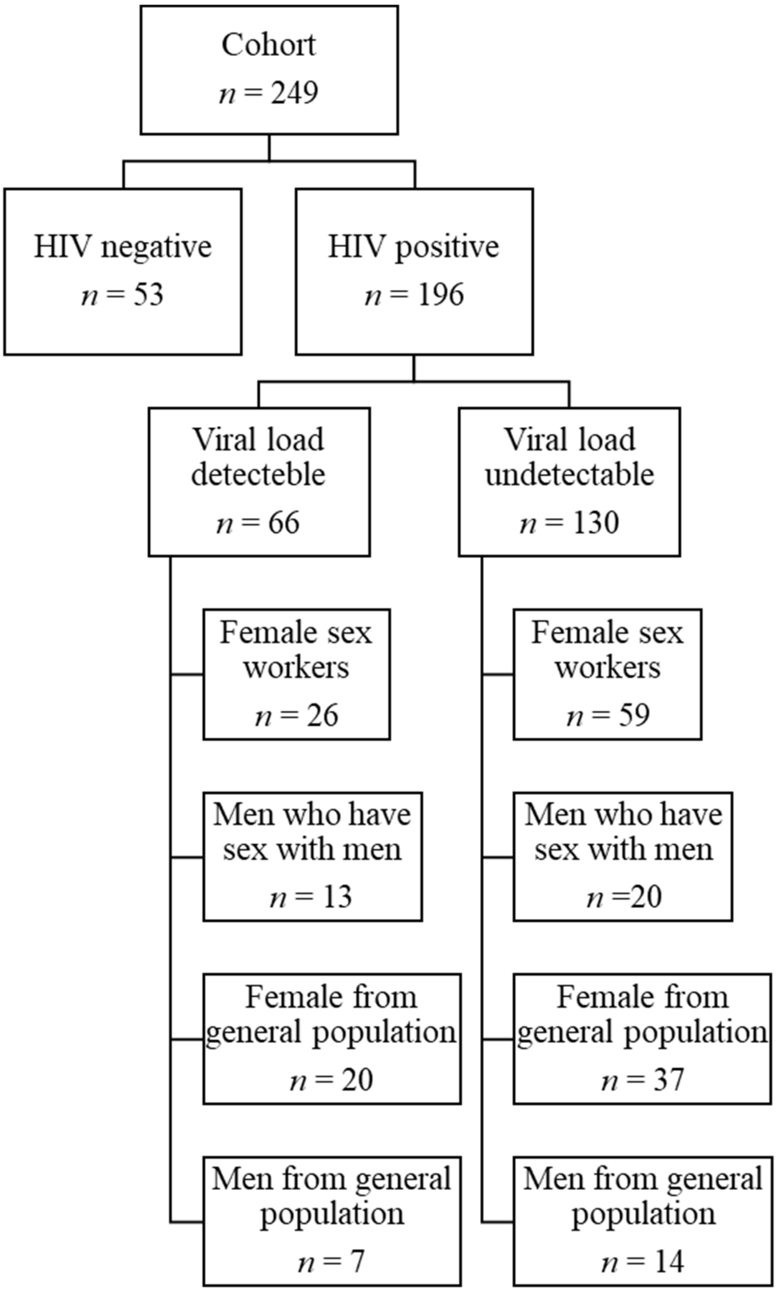

Figure S1. Flowchart of the participants (*n* = 249)

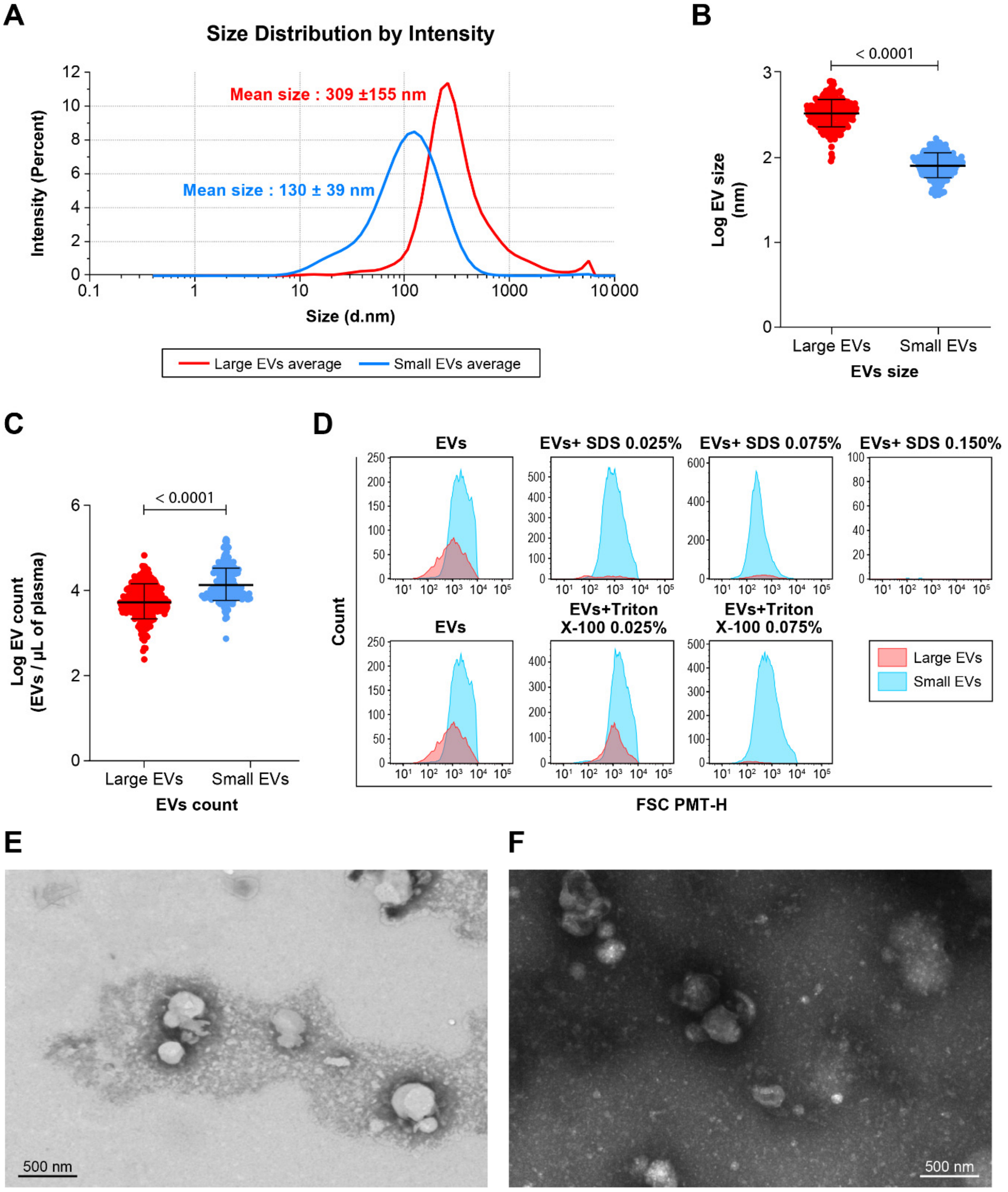

Figure S2. Characterization of plasma extracellular vesicles
EVs purified from pooled plasma were distinguished as large or small based on size distribution measured by dynamic light scattering (**A** and **B**) and counting by flow cytometry (**C**). The detergent lysis resistance of EVs category was performed (**D**). Transmission electron micrographs show visually the differences in typical large and small EV size (**E** and **F**). The size category and count comparisons are based on paired t tests.

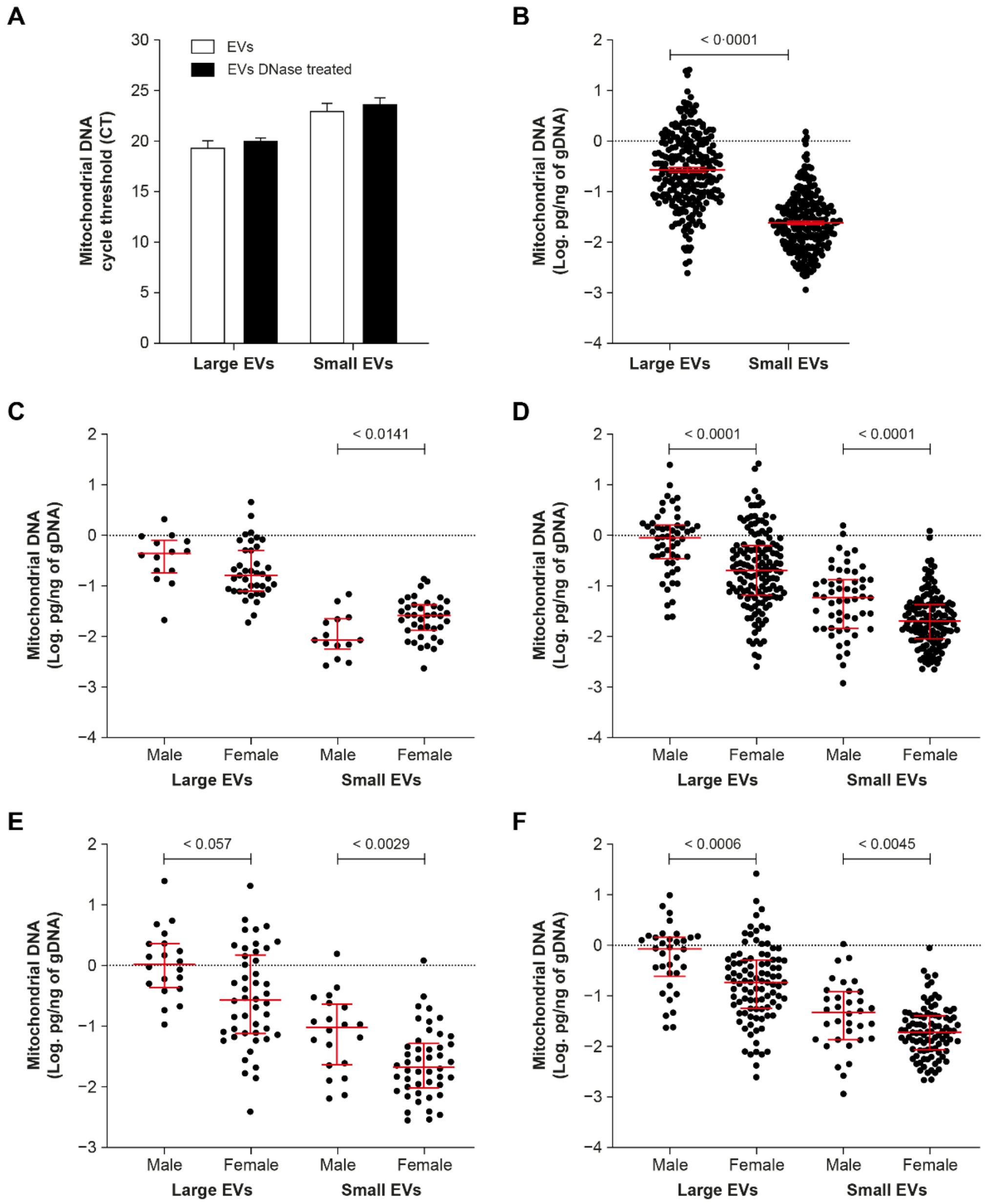

Figure S3. Mitochondrial DNA content of large and small extracellular vesicles in plasma obtained from all participants in the study.

(**A**) The impact of DNase treatment on the quantitative analysis of EV-borne mtDNA; (**B**) Mitochondrial DNA abundance in large and small EVs of all participants; Gender bias of mtDNA abundance in large and small EVs in uninfected participants (**C**) and HIV+ participants (**D**); Mitochondrial DNA abundance in large and small EVs of HIV+ participants with detectable viral load (E) and no detectable viral load (**F**). In graphs, the dots represent individual value and lines the geometric mean with geometric standard deviation factor or mean with standard error of mean. The comparisons are based on unpaired *t* tests.

Table S1. Comparison of large and small EVs mtDNA content in participants groups.

Groups of Participants	EVs mtDNA Content (Log 10 copies) Median (IQR)		*p* Value
	Large Evs	Small Evs	
Control participants (n=53)	-0.70 (-1.03– -0.19)	-1.68 (–2.09– –1.41)	<0.0001
Viremic participants (n=66)	0.03 (0.01–0.11)	-0.30 (-0.99- 0.25)	<0.0001
Viremic participants ART treated (n=51)	-0.54 (-1.09–0.14)	-1.65 (-2.00–-1.07)	<0.0001
Viremic participants tenofovir treated (n=32)	-0.34 (-1.05–0.21)	-1.60 (–1.99– -0.97)	<0.0001
ART naïve viremic (n=15)	0.17 (-0.22-0.64)	-1.31 (–1.54– -0.67)	<0.0001
Undetectable ART treated (n=118)	-0.61 (–1.14– –0.14)	-1.68 (–2.05– -1.29)	<0.0001
Undetectable tenofovir treated (n=80)	-0.52 (-1.04–0.03)	-1.65 (-1.97--1.12)	<0.0001
Female sex worker undetectable (n=58)	-0.86 (–1.39– –0.44)	-1.69 (–2.05– –1.34)	<0.0001
Female sex worker undetectable tenofovir treated (n=33)	-0.86 (–1.26– –0.53)	-1.71 (–2.56– –1.38)	<0.0001
Female sex worker viremic (n=26)	-0.56 (-1.10–0.03)	-1.55 (-1.93- -1.11)	<0.0001
Female sex worker viremic tenofovir treated (n=12)	-0.71 (–1.11– –0.03)	-1.67 (-1.96- -1.09)	0.0070
MSM undetectable tenofovir treated (n=20)	-0.06 (-0.52-0.14)	-1.07 (–1.58– –0.71)	<0.0001
MSM viremic (n=13)	-0.07 (-0.34-0.15)	-0.93 (–1.28– –0.56)	0.0002
MSM viremic tenofovir treated (n=9)	-0.07 (-0.53-0.25)	-0.93 (–1.08– –0.56)	0.0006
Female from general population undetectable (n=31)	-0.55 (–1.08– –0.06)	-1.88 (–2.24– –1.56)	<0.0001
Female from general population undetectable tenofovir treated (n=20)	-0.55 (–1.08– –0.06)	-1.88 (–2.24– –1.56)	<0.0001
Female from general population viremic (n=20)	-0.62 (-1.24–0.32)	-1.78 (–2.14– –1.38)	<0.0001
Female from general population viremic tenofovir treated (n=8)	-0.91 (-1.21–0.00)	-2.06 (–2.38– –1.87)	0.0008
Men from general population undetectable (n=9)	-0.42 (-1.28–0.32)	-1.86 (–2.37– –1.39)	0.0016
Men from general population undetectable tenofovir treated (n=7)	-0.06 (-0.95-0.48)	-1.98 (–2.40– –1.24)	0.0015
Men from general population viremic (n=7)	0.36 (-0.42-0.73)	-1.86 (–2.13– –1.07)	0.0002
men from general population viremic tenofovir treated (n=3)	0.36 (0.23-0.68)	-1.61 (–2.14– –1.07)	0.0386

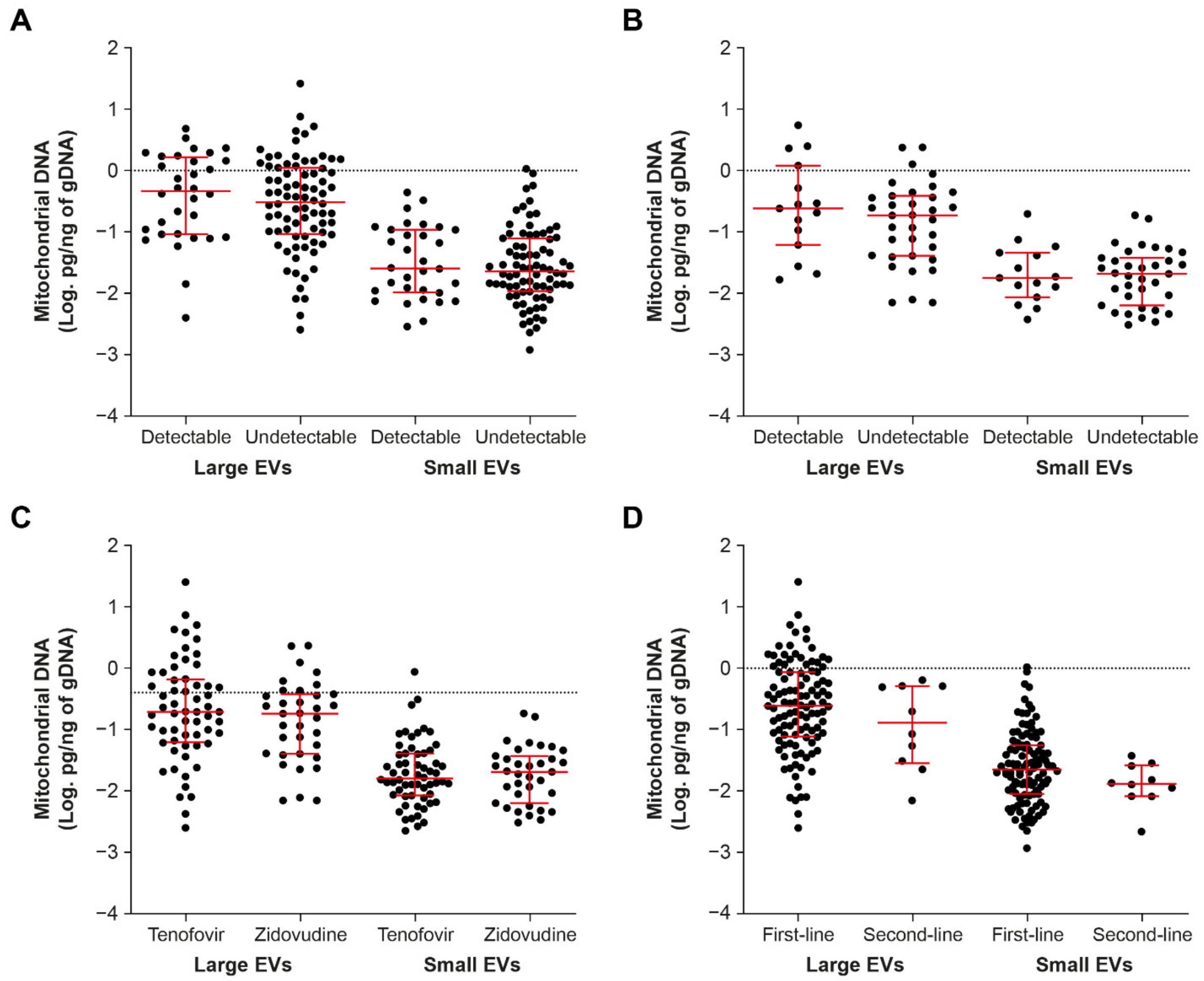

Figure S4. Mitochondrial DNA in large and small EVs in patients treated with Tenofovir (**A**) or with Zidovudine (**B**); in ART-treated patients excluding men who have sex with men (**C**) and according to NRTI-NNRTI order, that is, NRTI as first line or second line of treatment (**D**). In graphs, the dots represent individual value and lines the geometric mean with geometric standard deviation factor. Group comparisons are based on unpaired t tests.

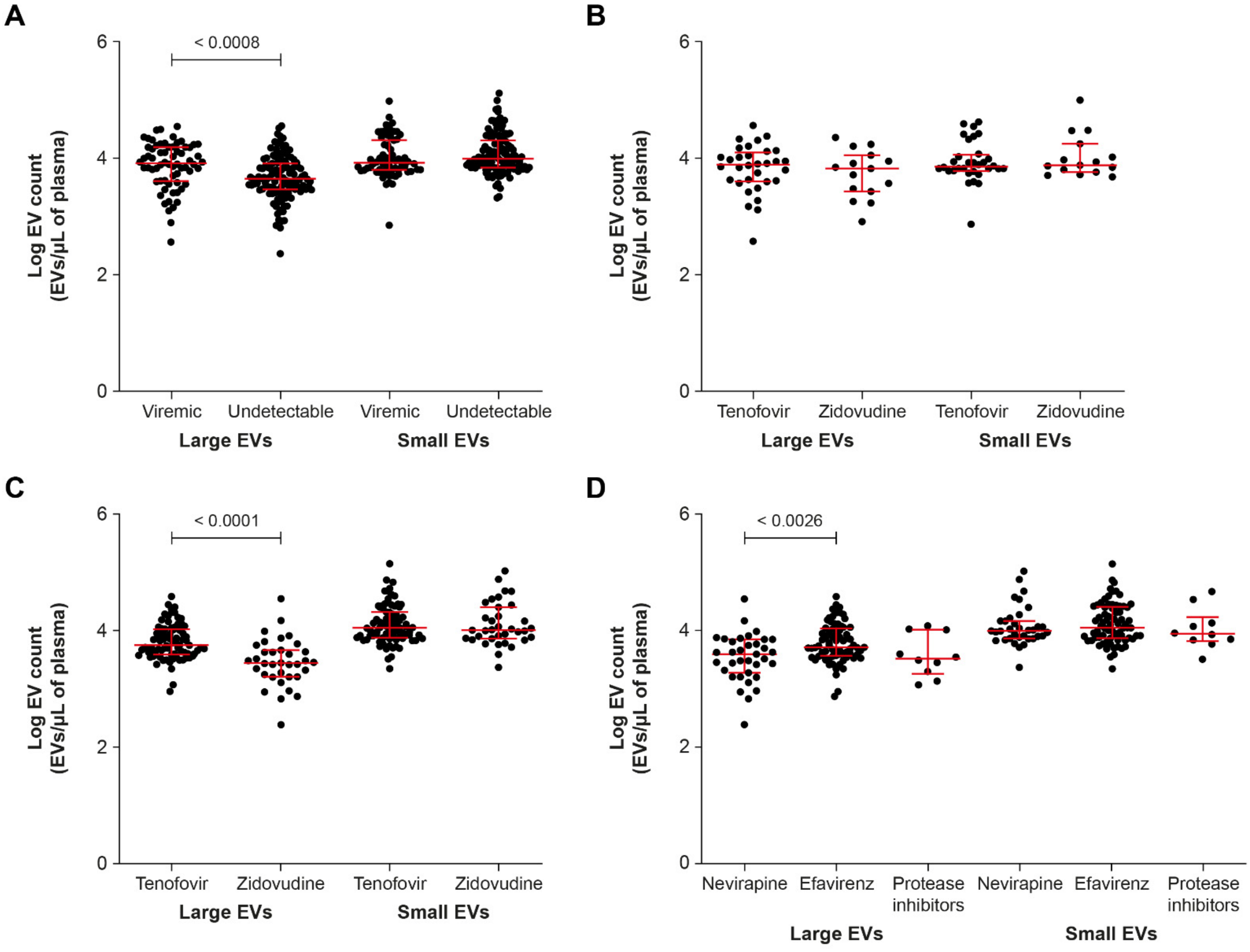

Figure S5. Large and small EV counts by cytometry in ART-treated participants with detectable and undetectable viremia (**A**); in Zidovudine and Tenofovir treated participants with detectable (**B**) and undetectable (**C)** viremia; after treatment with Nevirapine, Efavirenz and protease inhibitors (**D**). In graphs, the dots represent individual value and lines the geometric mean with geometric standard deviation factor. Group comparisons are based on t tests and ordinary one-way ANOVA with Tukey's multiple comparisons tests.

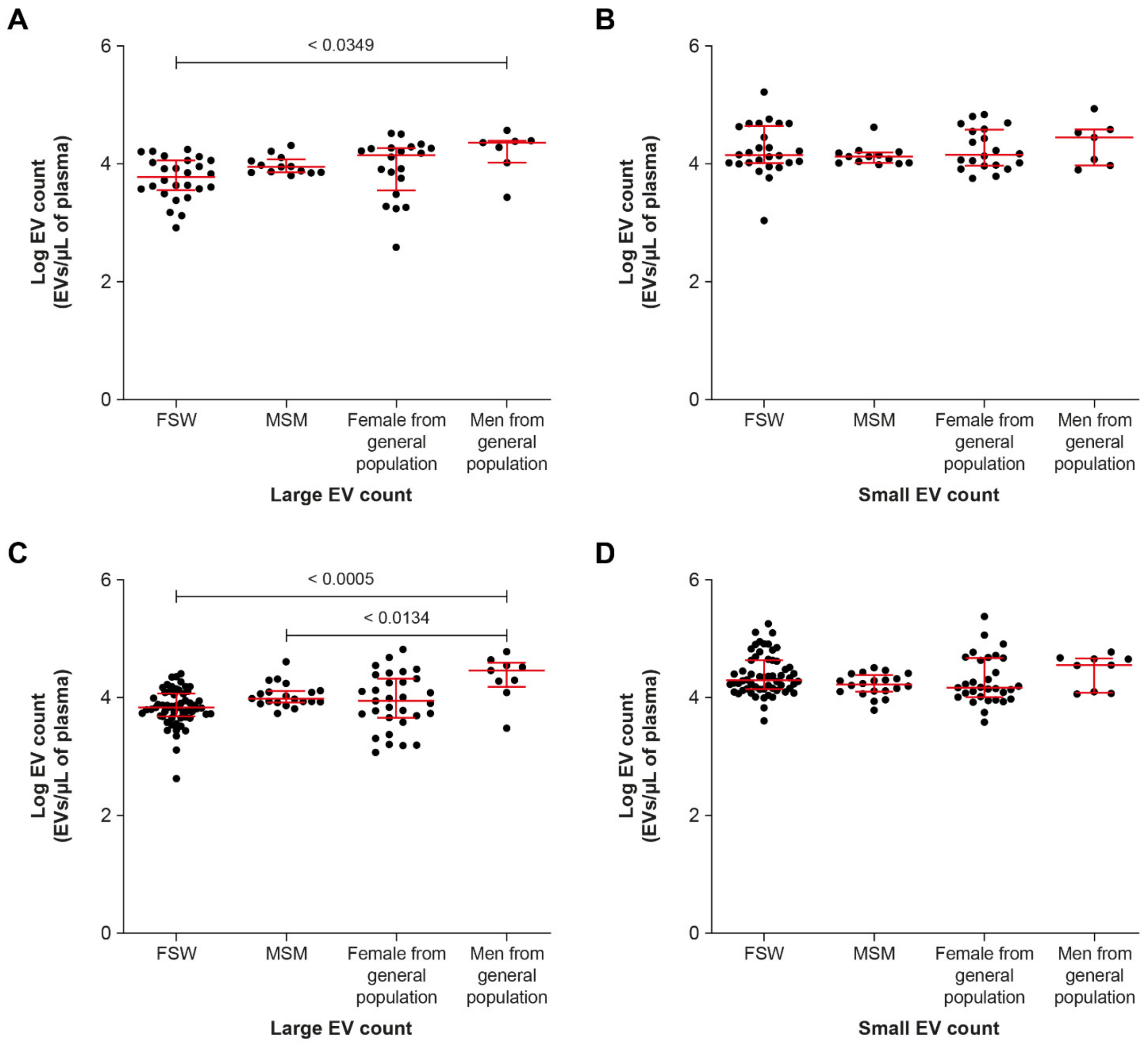

Figure S6. Large and small EV counts by cytometry in participant subgroups. **A**, **B**: viremic; **C**, **D**: non-viremic ART-treated participants. FSW = female sex workers; MSM = men who have sex with men. In graphs, the dots represent individual value and lines the geometric mean with geometric standard deviation factor. An ordinary one-way ANOVA with Tukey's multiple comparisons test was used to test for significant differences between groups.

Table S2. Correlation between mitochondrial DNA in large EVs and participant clinical parameters and other measurements

	HIV– (control) *n* = 53		HIV+ *n* = 196		HIV+ ART > 6 months, not viremic, *n* = 118		HIV+ ART > 6 months, viremic, *n* = 51		HIV+ ART-naïve, viremic, *n* = 15	
	**r (95% CI)**	**P value**	**r (95% CI)**	**P value**	**r (95% CI)**	**P value**	**r (95% CI)**	**P value**	**r (95% CI)**	**P value**
Age (years)	0.06 (-0.20 – 0.32)	0.6800	-0.23 (-0.36 – 0.09)	0.0013	-0.16 (-0.34 – 0.01)	0.0727	-0.47 (-0.66 – -0.23)	0.0005	0.15 (-0.39 – 0.61)	0.5953
Years HIV+	-	-	-0.29 (-0.41 – -0.55)	<0.0001	-0.30 (-0.46 – 0.13)	0.0009	-0.37 (-0.59 – -0.11)	0.0071	-0.09 (-0.57 – 0.44)	0.7599
Years on ART	-	-	-0.28 (-0.41 – 0.13)	0.0003	-0.24 (-0.40 – -0.06)	0.0096	-0.35 (-0.57 – -0.09)	0.0110	-	-
CD4 (cells/μL)	0.09 (-0.19-0.35)	0.5382	-0.02 (-0.16 – 0.12)	0.8189	-0.08 (-0.26 – 0.10)	0.3910	-0.01 (-0.28 – 0.27)	0.9563	0.31 (-0.24 – 0.71)	0.2603
CD8 (cells/μL)	0.15 (-0.13 – 0.40)	0.2908	-0.03 (-0.17 – 0.10)	0.6546	-0.04 (-0.22 – 0.14)	0.6560	-0.02 (-0.30 – 0.25)	0.8567	0.16 (-0.38 – 0.62)	0.5748
CD4/CD8 ratio	-0.15 (-0.40 – 0.13)	0.2882	-0.01 (-0.15 – 0.13)	0.8813	-0.05 (-0.23 – 0.13)	0.5945	0.02 (-0.25 – 0.29)	0.8789	0.10 (-0.44 – 0.58)	0.7310
Viral load (log_{10} copies)	-	-	0.03 (-0.21 – 0.27)	0.800	-	-	0.00 (-0.27 – 0.27)	0.9991	-0.33 (-0.72 – 0.22)	0.2279
Large EV count (per μL)	0.46 (0.22 – 0.65)	0.0005	0.51 (0.40 – 0.61)	< 0.0001	0.47 (0.32 – 0.60)	< 0.0001	0.50 (0.26 – 0.68)	0.0002	0.37 (-0.17 – 0.74)	0.1741

Table S3. Correlation between mitochondrial DNA in small EVs and participant clinical parameters and other measurements

	HIV– (control) *n* = 53		HIV+ *n* = 196		HIV+ ART > 6 months, not viremic, *n* = 118		HIV+ ART > 6 months, viremic, *n* = 51		HIV+ ART-naïve, viremic, *n* = 15	
	r (95% CI)	P value	r (95% CI)	P value	r (95% CI)	P value	r (95% CI)	P value	r (95% CI)	P value
Age (years)	0.26 (0.01 – 0.50)	0.0597	- 0.15 (-0.29 – 0.01)	0.0341	-0.14 (-0.32 – 0.04)	0.1178	-0.24 (-0.48 – 0.04)	0.0877	-0.05 (-0.55 – 0.47)	0.8490
Years HIV+	-	-	-0.20 (-0.33 – -0.06)	0.0044	-0.20 (-0.37 – -0.02)	0.0304	-0.11 (-0.38 – 0.17)	0.4255	-0.46 (-0.79 – 0.07)	0.0857
Years on ART	-	-	-0.17 (-0.31 – -0.02)	0.0251	-0.25 (-0.41 – 0.07)	0.0067	0.00 (-0.27 – 0.28)	0.9972	-	-
CD4 (cells/μL)	0.36 (0.10 – 0.58)	0.0079	-0.01 (-0.13 – 0.15)	0.8695	-0.02 (-0.20 – 0.16)	0.8075	0.20 (-0.08 – 0.45)	0.1569	-0.27 (-0.69 – 0.28)	0.3319
CD8 (cells/μL)	0.12 (-0.16 – 0.38)	0.4064	-0.03 (-0.17 – 0.11)	0.6361	0.00 (-0.18 – 0.18)	0.9829	-0.05 (-0.32 – 0.22)	0.7051	0.06 (-0.46 – 0.55)	0.8295
CD4/CD8 ratio	0.22 (-0.05 – 0.46)	0.1156	-0.05 (-0.09 – 0.18)	0.5226	-0.01 (-0.19 – 0.16)	0.8696	0.26 (-0.02 – 0.50)	0.0649	-0.18 (-0.63 – 0.37)	0.5293
Viral load (log_{10} copies)	-	-	-0.01 (-0.26 – 0.23)	0.9078	-	-	-0.12 (-0.38 – 0.16)	0.4153	0.03 (-0.49 – 0.53)	0.9203
Small EV count (per μL)	-0.19 (-0.44 – 0.09)	0.1812	0.07 (-0.07 – 0.20)	0.3460	0.03 (-0.15 – 0.21)	0.7558	0.05 (-0.22 – 0.32)	0.7044	-0.09 (-0.57 – 0.44)	0.7552

Table S4. Diagnostic performance of the mtDNA abundance in large and small EVs for distinguishing viremic and non-viremic ART-treated participants, based on receiver operating characteristic curve analysis

		**All participants**	**Female sex workers**	**Men who have sex with men**	**Females, general population**	**Males, general population**
		**Area under curve (95%CI)**				
**Viremic**	**Large EVs**	0.72 * (0.53 – 0.91) *n* = 66	0.66 (0.43 – 0.89) *n* = 26	0.79 * (0.59 – 1.0) *n* = 13	0.67 (0.45 – 0.88) *n* = 20	0.87 * (0.69 – 1.0) *n* = 7
	**Small EVs**	0.64 (0.49 – 0.80) *n* = 66	0.63 (0.44 – 0.83) *n* = 26	0.96 *** (0.88 – 1.0) *n* = 13	0.50 (0.28 – 0.73) *n* = 20	0.54 (0.24 – 0.84) *n* = 7
**CD4/CD8 cell ratio < 1**	**Large EVs**	0.64 (0.42 – 0.85) *n* = 85	0.56 (0.31 – 0.82) *n* = 39	0.79 * (0.58 – 1.0) *n* = 11	0.67 (0.45 – 0.88) *n* = 25	0.70 (0.46 – 0.94) *n* = 10
	**Small EVs**	0.55 (0.38 – 0.73) *n* = 85	0.55 (0.36 – 0.75) *n* = 39	0.83 * (0.64 – 1.0) *n* = 11	0.53 (0.31 – 0.76) *n* = 25	0.52 (0.25 – 0.79) *n* = 10
**≥ 500 CD8 cells/μL**	**Large EVs**	0.62 (0.39 – 0.85) *n* = 94	0.57 (0.31 – 0.82) *n* = 46	0.72 (0.47 – 0.96) *n* = 11	0.65 (0.43 – 0.88) *n* = 28	0.68 (0.42 – 0.93) *n* = 9
	**Small EVs**	0.54 (0.36 – 0.72) *n* =94	0.56 (0.37 – 0.75) *n* = 46	0.80 * (0.60 – 1.0) *n* = 11	0.57 (0.34 – 0.79) *n* = 28	0.52 (0.24 – 0.80) *n* = 9

List of abbreviations

EVs: Extracellular vesicles

mtDNA: Mitochondrial DNA

ART: Antiretroviral therapy

PLWH: Persons or people living with HIV

HIV: human immunodeficiency virus

NRTIs: Nucleoside-analog reverse transcriptase inhibitors

NNRTIs: Non-nucleoside reverse transcriptase inhibitors

PIs: Protease inhibitors

INIs: integrase inhibitors

TLRs: Toll-like receptors

AIM-2: Absent in melanoma 2

cGAS: Cyclic GMP-AMP Synthase

CHU : *Centre Hospitalier Universitaire*
